# Genome-wide DNA methylation profiles in Tibetan and Yorkshire pigs under high-altitude hypoxia

**DOI:** 10.1186/s40104-019-0316-y

**Published:** 2019-02-05

**Authors:** Bo Zhang, Dongmei Ban, Xiao Gou, Yawen Zhang, Lin Yang, Yangzom Chamba, Hao Zhang

**Affiliations:** 1 National Engineering Laboratory for Animal Breeding, Beijing Key Laboratory for Animal Genetic Improvement, China Agricultural University, Beijing, 100193 China; 2 College of Animal Science and Technology, Yunnan Agricultural University, Kunming, 650201 China; 3 College of Animal Science, Tibet Agriculture and Animal Husbandry University, Linzhi, 860000 Tibet China

**Keywords:** DNA methylation, Hypoxic adaptation, MeDIP-seq, Tibetan pig

## Abstract

**Background:**

Tibetan pigs, which inhabit the Tibetan Plateau, exhibit distinct phenotypic and physiological characteristics from those of lowland pigs and have adapted well to the extreme conditions at high altitude. However, the genetic and epigenetic mechanisms of hypoxic adaptation in animals remain unclear.

**Methods:**

Whole-genome DNA methylation data were generated for heart tissues of Tibetan pigs grown in the highland (TH, *n* = 4) and lowland (TL, *n* = 4), as well as Yorkshire pigs grown in the highland (YH, *n* = 4) and lowland (YL, *n* = 4), using methylated DNA immunoprecipitation sequencing.

**Results:**

We obtained 480 million reads and detected 280679, 287224, 259066, and 332078 methylation enrichment peaks in TH, YH, TL, and YL, respectively. Pairwise TH vs. YH, TL vs. YL, TH vs. TL, and YH vs. YL comparisons revealed 6829, 11997, 2828, and 1286 differentially methylated regions (DMRs), respectively. These DMRs contained 384, 619, 192, and 92 differentially methylated genes (DMGs), respectively. DMGs that were enriched in the hypoxia-inducible factor 1 signaling pathway and pathways involved in cancer and hypoxia-related processes were considered to be important candidate genes for high-altitude adaptation in Tibetan pigs.

**Conclusions:**

This study elucidates the molecular and epigenetic mechanisms involved in hypoxic adaptation in pigs and may help further understand human hypoxia-related diseases.

**Electronic supplementary material:**

The online version of this article (10.1186/s40104-019-0316-y) contains supplementary material, which is available to authorized users.

## Background

Epigenetic modification, including DNA methylation and histone modification, results in long-term regulations of gene expression [1]. In mammals, DNA methylation predominantly occurs at the C-5 position of cytosine in CpG dinucleotides, which are sparsely distributed in the genome, with the exception of short genomic regions called CpG islands (CGIs) [2, 3]. CpG methylation stabilizes the chromatin structure and may regulate the accessibility of these DNA regions for the transcriptional machinery [4]. Short-term severe hypoxia results in long-lasting changes in the genome-wide DNA methylation status, some of which may be highly correlated with the transcriptional modulation of several genes involved in functional pathways [5]. DNA methylation also plays an important role in regulating the transcription of hypoxia-response genes [6]. For example, DNA methylation in the promoter region of the estrogen receptor gene has been shown to increase under conditions of chronic hypoxia in the uterine arteries of pregnant sheep, thereby inhibiting gene expression [7]. Another previous study revealed significant genome-wide epigenetic differences between Ethiopians living at high and low altitudes [8]. Therefore, DNA methylation, an important epigenetic mechanism, may allow for the comprehensive profiling of hypoxic responses and adaptation in animals. Furthermore, as hypoxic stress is known to influence gene expression via the modification of DNA methylation, which has been studied with regard to tumor suppressor genes and cancer progression [9]; hypoxia has been shown to promote DNA hypermethylation in tumors [10] and alter gene expression in osteoblasts [11].

An important technical advance for analyzing genome-wide DNA methylation includes immunoprecipitation, which is used to enrich the genome portion containing either 5-methylcytosine or 5-hydroxymethylcytosine, depending on the antibody used, followed by high-throughput sequencing [12–14]. The methylated DNA immunoprecipitation (MeDIP)-based approach results in the enrichment of methylated DNA regions and can be combined with gene-by-gene polymerase chain reaction (PCR) detection and tiling microarrays [15–17]. MeDIP, in conjunction with high-throughput sequencing (MeDIP-seq), provides a genome-wide coverage technique that has successfully been used to profile global DNA methylation patterns in mammalian genomes [13] and in several tissues, including human breast cancer cells [18], peripheral blood mononucleocytes [19], and malignant nerve tumors [20].

Tibetan pigs inhabit the Tibetan Plateau and have a well-developed heart, which is better adapted to hypoxia than that of lowland pigs [21, 22]. A number of rapidly evolving and positively selected genes have been reported for Tibetan pigs based on high-throughput sequencing [23–27], and several important candidate genes for high-altitude adaptation, including genes involved in angiogenesis, ATP binding, and glucose metabolic processes as well as important pathways such as the hypoxia-inducible factor 1 (HIF-1) signaling pathway, have been revealed by comparative transcriptomic and proteomic analyses of Tibetan pig heart tissues [28]. However, the mechanisms involved in the regulation of gene expression at high altitude remain unclear. In the present study, we used MeDIP-seq to examine the genome-wide DNA methylation landscape in the heart tissues of Tibetan and Yorkshire pigs grown in high- and lowland areas to evaluate whether DNA methylation regulates the expression of certain key genes, potentially involved in high-altitude adaptation. Our results may further elucidate the regulation of genes involved in adaptation to high altitude in other species.

## Methods

### Sample preparation and DNA isolation

Castrated boars from four groups, which were conceived, born, and raised in either highland (Linzhi, 3000 m) or lowland (Beijing, 100 m) areas, including Tibetan highland pigs (TH, *n* = 9), Yorkshire highland pigs (YH, *n* = 9), Tibetan lowland pigs (TL, *n* = 9), and Yorkshire lowland pigs (YL, *n* = 9), were slaughtered and sampled at six months of age. The immigrant groups (YH and TL) descended from populations that had migrated to their raising places approximately three years earlier and had been bred for one generation. Prior to the experiment, all pigs were vaccinated and regularly dewormed during feeding. The feeding method included free access to drink water and feed. The same feeding method was maintained for different pigs at the same elevation. DNA was isolated from heart tissue samples using the TIANamp Genomic DNA Kit (Tiangen Biotech, Beijing, China).

### Library construction and sequencing

DNA (1 μg) was sonicated using a Covaris sonication system (Covaris, Woburn, MA, USA) according to previously established parameters [19] to obtain approximately 250-bp fragments. To end-repair DNA fragments, “adenine” nucleotides were added to the 3′ end and ligated to Agencourt® AMPure XP beads (Beckman Coulter, Brea, CA, USA). The Magnetic Methylated DNA Immunoprecipitation Kit (Diagenode, Liège, Belgium) was used to perform the methylation analysis by MeDIP. DNA enriched by MeDIP was amplified by PCR using the TruSeq DNA sample preparation kit and PCR Prep Box (Illumina, San Diego, CA, USA). Clustering of the index-coded samples was performed on a cBot Cluster Generation System using the cBot user guide (Illumina). After cluster generation, the libraries were sequenced using a HiSeq 2500 platform (Illumina), and 50 bp long single-end reads were generated. Four biological replicates were included in each pig group examined.

### MeDIP-seq data analysis

Raw reads obtained by MeDIP-seq were preprocessed using FASTX (version 0.0.13) (http://hannonlab.cshl.edu/fastx_toolkit/index.html) by removing reads containing adapters, low-quality reads, and reads shorter than 20 bp. The remaining (clean reads were then mapped to the pig reference genome of *Sus scrofa* 10.2.69 (ftp://ftp.ensembl.org/pub/release-69/fasta/sus_scrofa/dna/Sus_scrofa.Sscrofa10.2.69.dna.toplevel.fa.gz) using Bowtie (version 0.12.8). Methylation enrichment peaks were counted (DiffScore ≥50) using MACS (version 1.4) [29]. The MEDIPS package [30] was used to identify differentially-methylated regions [DMRs; window size = 500 bp, fold change ≥1.33 or ≤ 0.75, *P* ≤ 0.001]. Differentially methylated genes (DMGs) within DMRs were annotated using the UCSC database (https://genome.ucsc.edu/). According to the positions of the DMRs, we annotated related genomic elements as follows: promoter, 5′- untranslated region (UTR), coding sequence (CDS), 3′-UTR, exon, intron, and transcription termination region (TTR). The promoter region was defined as the 5000 bp sequence upstream of the transcription initiation site, and TTR was defined as the 5000 bp downstream of the transcription termination site. The CGI regions included “shores” (2000 bp flanking the CGIs) and “shelves” (2000 bp beyond CpG shores), while “open sea” regions were located outside CGIs. DMGs were classified into functional categories using Gene Ontology (GO) terms and Kyoto Encyclopedia of Genes and Genomes (KEGG) pathway annotations using DAVID (https://david.ncifcrf.gov/home.jsp) [31].

### Bisulfite sequencing PCR to validate MeDIP-seq

Approximately 2 μg of DNA from each sample was subjected to bisulfite conversion with the EpiTect Bisulfite Kit (Qiagen, Frankfurt, Germany) according to the manufacturer’s protocol. Using MethPrimer (http://www.urogene.org/cgi-bin/methprimer/methprimer.cgi), we designed five pairs of bisulfite sequencing PCR (BSP) primers for five DMGs, including the branched-chain keto acid dehydrogenase E1 subunit beta (*BCKDHB*), epoxide hydrolase 2 (*EPHX2*), glutamic-oxaloacetic transaminase 2 (*GOT2*), retinoid X receptor gamma (*RXRG*), and ubiquitin D (*UBD*) genes, to confirm the results of MeDIP-seq (Additional file 1). All PCR products were gel-purified using a gel purification kit (BioTeke Corporation, Beijing, China), then ligated into the pMD18-T vector (TaKaRa, Kusatsu, Shiga, Japan), and transformed into *Escherichia coli* DH5α competent cells (Beijing Biotech Co., Ltd., Beijing, China). Eight to twelve monoclones from each individual were sequenced by Sangon Biotech Co., Ltd. (China). The acquired sequences were processed using the online tool QUMA (QUantification tool for Methylation Analysis) [32]. Samples from five individuals (differing from the four individuals whose samples were used for MeDIP-seq) from each group were used for BSP. Differences in DNA methylation for each gene were analyzed using Fisher’s exact test, and differences were considered statistically significant at *P* < 0.05.

### Quantitative real-time PCR

RNA extraction and cDNA synthesis were performed using the RNAprep Pure Kit (for tissue) and FastKing RT Kit (Tiangen Biotech). The hypoxanthine phosphoribosyltransferase (*HPRT*) gene was used as an internal control. The primers for *HPRT* and the five DMGs (*BCKDHB*, *EPHX2*, *GOT2*, *RXRG*, and *UBD*) are listed in Additional file 2. Quantitative real-time PCR (qPCR) was performed using SuperReal PreMix Plus (Tiangen Biotech) and a CFX96 real-time system (Bio-Rad, CA, USA). A cDNA pool of all samples was used for calibration, and three replications of each sample were performed. Samples from eight individuals from each of the four groups were used for qPCR. Gene expression levels were calculated using the 2^−ΔΔCt^ method [33] and analyzed with one-way analysis of variance using SAS (version 9.1) (SAS Institute, Inc., Cary, NC, USA). Graphs were prepared using SigmaPlot (version 10.0) (Systat Software, San Jose, CA, USA), and data are presented as the mean ± standard error of the mean.

## Results

### Summary of MeDIP-seq data

Approximately 480 million reads were generated by MeDIP-seq from 16 heart tissue samples (*n* = 4 per group) of TH, YH, TL, YL (approximately 30 million reads per individual). Approximately 85% of these reads were mapped to the porcine reference genome, and approximately 55% were uniquely mapped to specific regions (Additional file 3). The MeDIP-seq reads were distributed in all chromosomes (1–18 and X), with many peak shapes in the four groups of pigs. The density of normalized reads mapped to the proximal and distal regions of chromosomes was higher than that of reads mapped to other regions in all individuals. Similar uneven distributions have been observed in previous studies [34].

### Methylation peaks

Scanning of the MeDIP-seq reads revealed 280679, 287224, 259066, and 332078 highly methylated regions (peaks) in the TH, YH, TL, and YL samples, respectively (Table 1). The lengths of the peaks were 500–1200 bp and averaged 943–1027 bp in the four groups (Additional file 4: Figure S1). The peak regions covered 11.99%, 12.82%, 10.47%, and 15.06% of the genome in TH, YH, TL, and YL, respectively. Most of the methylated peaks mapped to intergenic regions (236438–302779 peaks), followed by intron regions (13951–18130 peaks), while only 3024–3897, 3129–3934, and 2524–3338 peaks mapped to promoters, exons, and TTRs, respectively (Additional file 4: Figure S2).

**Table 1:** Information on methylation peaks in the four groups of pigs

Samples	Number of peaks	Mean peak length	Median peak length	Total peak length	Peak covered size in genome, %
TH	280678	1199.23	995	336598369	11.99
YH	287224	1253.43	1027	360015941	12.82
TL	259066	1135.53	943	294176561	10.47
YL	332078	1273.89	1045	423031970	15.06

*TH* Tibetan highland pig, *YH* Yorkshire highland pig, *TL* Tibetan lowland pig, *YL* Yorkshire lowland pig

### Differentially-methylated regions among the four groups

Comparison of TH vs. YH and TL vs. YL resulted in 6829 and 11997 DMRs respectively, which revealed the breed effects in highland and lowland environments. Comparison of TH vs. TL and YH vs. YL resulted in 2826 and 1286 DMRs, respectively, which revealed the effects of hypoxia on DNA methylation (Additional file 5). The number of DMRs between the two breeds (TH vs. YH or TL vs. YL) was higher than that between the two elevations (TH vs. TL or YH vs. YL; Table 2). Most DMRs were distributed in intergenic regions, followed by introns, TTRs, promoters, and CDS regions, while 5′- and 3′- UTRs contained few DMRs (Additional file 4: Figure S3). Furthermore, DMRs were mostly in open sea regions, followed by shelf, shore, and CGI regions (Additional file 4: Figure S4). Of the between-breed DMRs, much fewer (39.45% and 20.54% in high- and lowland comparison groups, respectively) were hypermethylated in Tibetan pig (Table 2). In comparison between the highland and lowland pigs of the same breed showed that Tibetan pigs had a high percentage (68.32%) and Yorkshire pigs had a low percentage (45.65%) of hypermethylated DMRs in the highland groups. Overall, the DNA methylation patterns of the two breeds differed in response to high-altitude hypoxia. We annotated 384, 619, 192, and 92 DMGs from DMRs identified by comparing TH vs. YH, TL vs. YL, TH vs. TL, and YH vs. YL, respectively (Table 2 and Additional file 5). Of these, 14 DMGs were common to the four comparisons groups, 18 were common to the TH vs. TL and YH vs. YL, while 183 DMGs were common to the TH vs. YH and TL vs. YL (Additional file 4: Figure S5).

**Table 2:** Numbers of DMRs revealed by four pairwise comparisons

Comparison groups	Total DMRs	Hypermethylated DMRs	Hypomethylated DMRs	DMGs
TH vs. YH	6829	2694 (39.45%)	4135 (60.55%)	384
TL vs. YL	11997	2464 (20.54%)	9533 (79.46%)	619
TH vs. TL	2828	1932 (68.32%)	896 (31.68%)	192
YH vs. YL	1286	587 (45.65%)	699 (54.35%)	92

*TH* Tibetan highland pig, *YH* Yorkshire highland pig, *TL* Tibetan lowland pig, *YL* Yorkshire lowland pig

### DMGs validation and gene expression

Five DMGs, *BCKDHB*, *EPHX2*, *GOT2*, *RXRG*, and *UBD*, which all exhibited higher levels of DNA methylation in TH than in YH, were selected to measure methylation at CpG sites, based on bisulfite sequencing and the expression levels detected by real-time fluorescence qPCR (Additional file 4: Figure S6). The methylated peaks and CpG sites evaluated were all within the intronic regions of the five genes. In the bisulfite sequencing experiment, the methylation of *BCKDHB*, *GOT2*, *RXRG*, and *UBD* was higher in TH than in YH (*P* < 0.05), while only *EPHX2* showed no significant difference in methylation between TH and YH (*P* > 0.05; Additional file 4: Figure S6 and Table 3). In the qPCR experiment, the gene expression levels of the five DEGs were lower in TH than in YH, which indicated that up-methylation in the intron regions down-regulated the expression of these genes (Additional file 4: Figure S5 and Additional file 6). The concordance between the data obtained by bisulfite sequencing and qPCR indicated that the DMGs identified by MeDIP-seq were credible.

**Table 3:** Methylation ratios of CpG sites and Fisher’s exact *P* values for the five selected genes

Gene		TH	YH	TL	YL	*P*-value in TH/YH	*P*-value in TL/YL	*P*-value in TH/TL	*P*-value in YH/YL
*BCKDHB*	Methylated	113	97	108	96	0.0029	0.0457	0.3329	1.0000
	Unmethylated	7	23	12	24				
	Methylated ratio	0.9417	0.8083	0.9000	0.8000				
*EPHX2*	Methylated	133	146	129	148	0.3200	0.0500	0.5200	0.9100
	Unmethylated	62	54	70	52				
	Methylated ratio	0.6821	0.7300	0.6482	0.7400				
*GOT2*	Methylated	104	108	96	96	0.0300	0.2463	1.0000	0.5085
	Unmethylated	0	6	0	3				
	Methylated ratio	1.0000	0.9474	1.0000	0.9697				
*RXRG*	Methylated	279	295	268	244	0.0160	0.7000	1.05E-05	0.0970
	Unmethylated	27	52	73	62				
	Methylated ratio	0.9118	0.8501	0.7859	0.7974				
*UBD*	Methylated	75	66	62	71	0.0483	0.1301	0.0098	0.3677
	Unmethylated	5	14	16	9				
	Methylated ratio	0.9375	0.8250	0.7949	0.8875				

### Functional annotation of DMGs

The main GO terms enriched in the 384 DMGs that were identified by TH vs. YH comparison and might be related to hypoxic adaptation were acetyl-CoA carboxylase activity, cellular response to hyperoxia, and relaxation of vascular smooth muscle, while the KEGG pathways included adipocytokine, insulin, forkhead box protein O (FoxO), AMP-activated protein kinase (AMPK) signaling, etc. The main GO terms enriched in the 619 DMGs that were identified by TL vs. YL comparison and that could be related to inherent genetic differences between the two breeds were glycogen biosynthetic process, relaxation of vascular smooth muscle, and NAD^+^ binding, while the KEGG pathways included biosynthesis of antibiotics and metabolic and peroxisome proliferator-activated receptor (PPAR) signaling pathways. The main GO terms enriched in the 192 DMGs that were identified by TH vs. TL comparison and might be related to hypoxic responses in Tibetan pigs were striated muscle tissue development, venous blood vessel morphogenesis, and glycosaminoglycan binding, while the KEGG pathways included PPAR and adipocytokine signaling and fatty acid metabolism. The main GO terms enriched in the 92 DMGs that were identified by YH vs. YL comparison and that could be related to responses to hypoxia in Yorkshire pigs were inhibin A complex, calcineurin–nuclear factor of activated T cells (NFAT) signaling cascade, and hemoglobin biosynthetic process, while the main KEGG pathway category was regulation of actin cytoskeleton (Additional file 7, Additional file 8, Fig. 1, and Fig. 2).

**Fig. 1:**
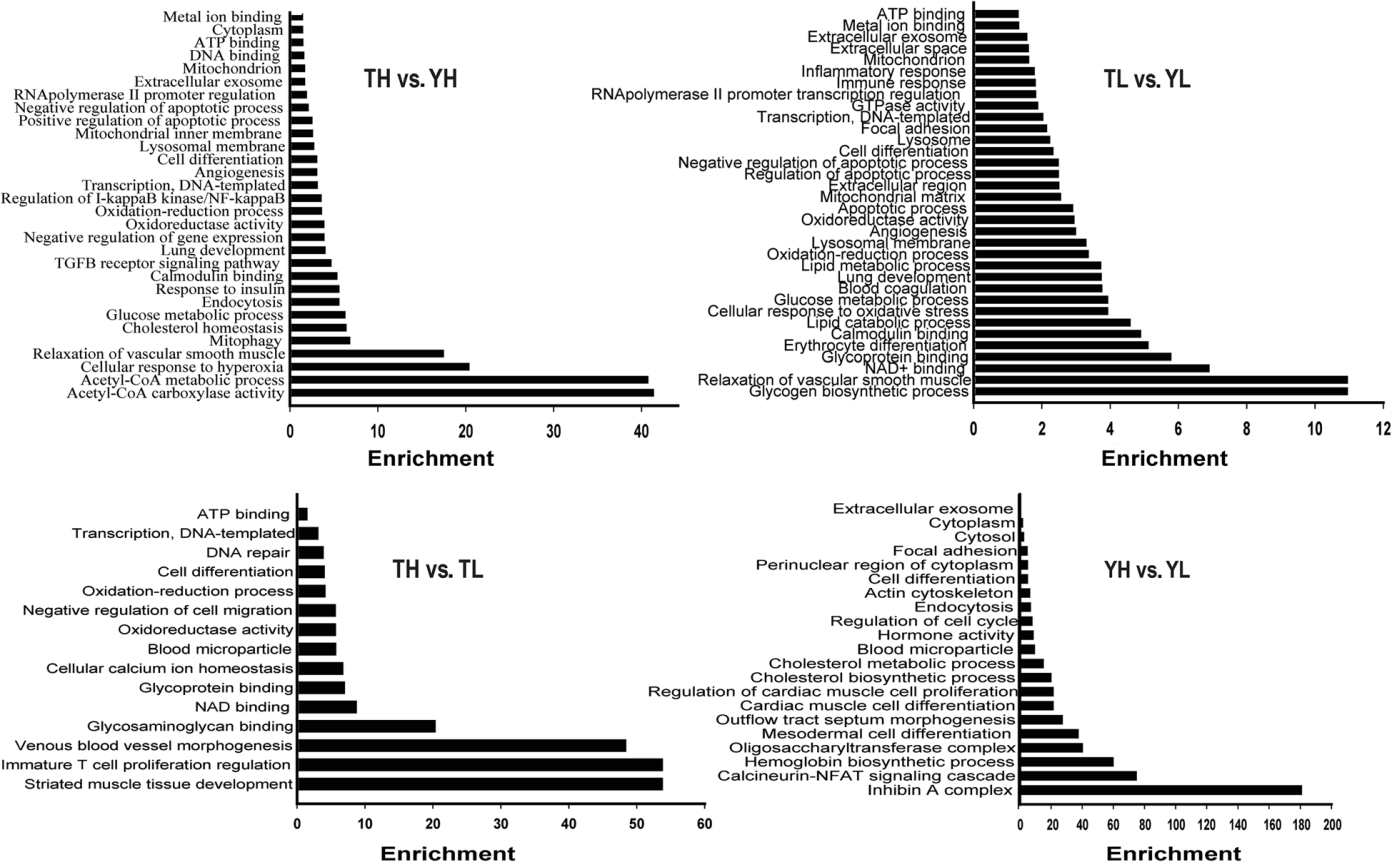
Gene Ontology (GO) terms enriched in DMGs identified by comparison of TH vs. YH, TL vs. YL, TH vs. TL and YH vs. YL. The *X*-axis indicates GO enrichment, and *Y*-axis indicates GO terms

**Fig. 2:**
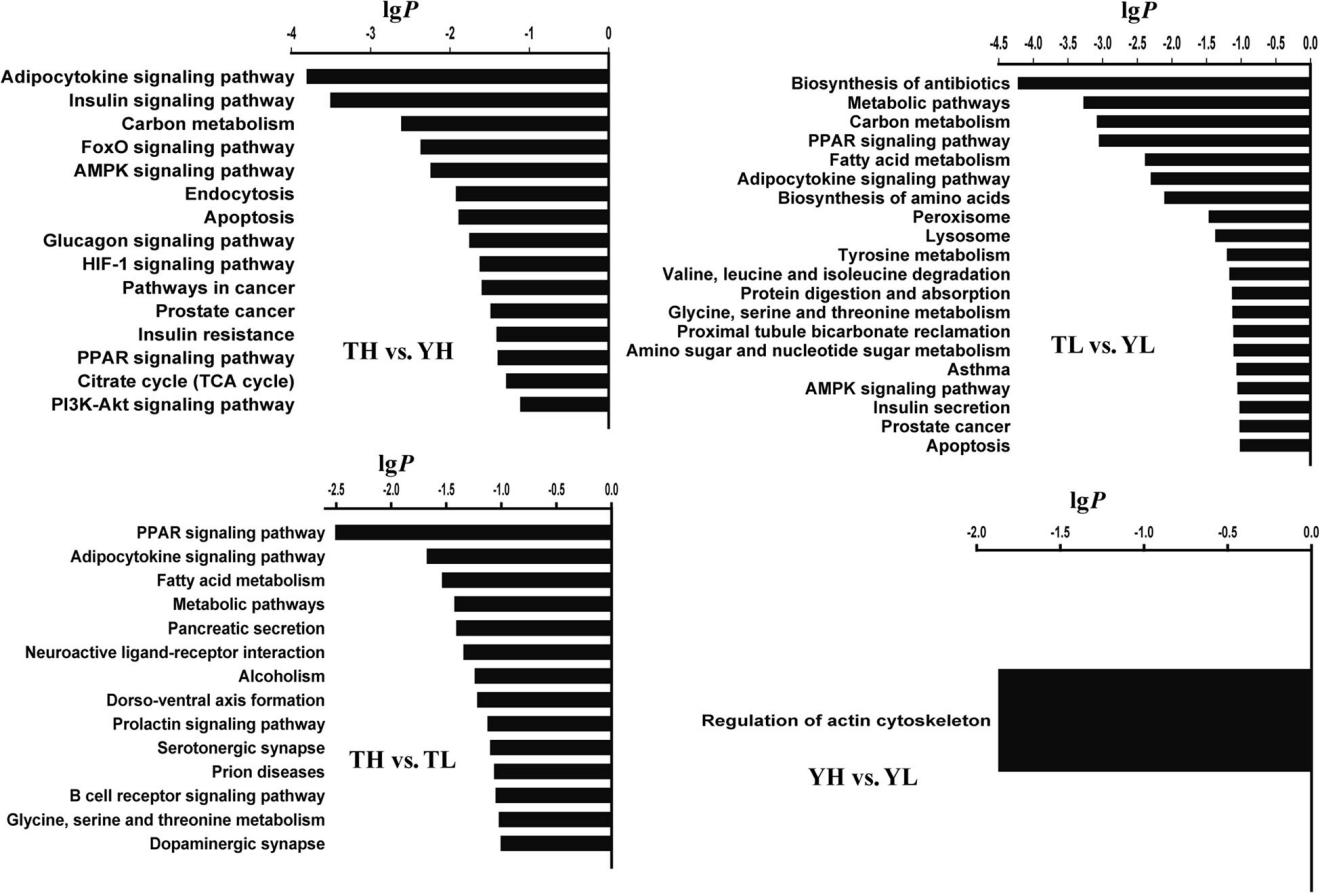
KyotoEncyclopedia of Genes and Genomes (KEGG) pathways enriched in DMGs identified by comparison of TH vs. YH, TL vs. YL, TH vs. TL and YH vs. YL. The *X*-axis indicates the pathway terms and *Y*-axis indicates KEGG pathway enrichment

We observed the overrepresentation of several GO terms and KEGG pathways (common in the TH vs. YH and TL vs. YL), which included angiogenesis (enriched with *FGFR2*, *EPAS*1, and *ANGPT2*), the AMPK signaling pathway (enriched with *IGF1R* and *FOXO1*), and cell differentiation (enriched by *ERG*, *GRB2*, *FOXO1*, and *ANGPT*2). DMGs between TH and YH were also enriched in several interesting pathways, which included HIF-1 signaling (*AKT3*, *ANGPT2*, *EIF4E2*, *HK2*, *ICA*, *IGF1R*, *IL6R*, and *MAP2K1*), pathways in cancer (*AKT3*, *EPAS1*, *FADD*, *FGFR2*, *GRB2*, *KIT*, *MAP2K1*, *STAT5B*, *FOXO1*, and *SMAD2*), phosphoinositide 3-kinase (PI3K/protein kinase B (also known as AKT) signaling (*FGFR2*, *MAP2K1*, *IL6R*, *KIT*, *IGF1R*, *ANGPT2*, *AKT3*, and *EIF4E2*), and insulin signaling (*MAP2K1*, *INPPL1*, *GRB2*, *HK2*, *ACACA*, *FOXO1*, *ACACB*, *PCK2*, *PPP1CB*, *PRKAR2A*, *PTPN1*, *AKT3*, and *EIF4E2*) (Additional file 8). The aforementioned pathways were all connected to the HIF-1 signaling pathway in common (https://www.kegg.jp/pathway/hsa04066) and could be involved in the regulation of oxygen balance in the tissues and cells of animals. Based on the functions of DMGs found among the four groups, 19 DMGs were identified, which were possibly related to high-altitude adaptation in Tibetan pigs (Table 4).

**Table 4:** Key differentially methylated genes possibly related to hypoxic adaption in Tibetan pigs

Gene	Description	TH vs. YH	TL vs. YL	TH vs. TL	YH vs. YL	Functional annotation
*AKT3*	AKT serine/threonine kinase 3	+	–	–	–	HIF-1 signaling pathway, VEGF signaling pathway, pathways in cancer
*ANGPT2*	Angiopoietin 2	+	+	+	–	Angiogenesis, HIF-1 signaling pathway
*CAT*	Catalase	+	–	–	–	Oxygen species, apoptosis process, mitochondrion
*CD38*	Cluster of differentiation 38	+	+	+	+	Calcium signaling pathway
*EIF4E2*	Eukaryotic translation initiation factor 4E family member 2	+	–	–	–	HIF-1 signaling pathway, mTOR signaling pathway, insulin signaling pathway
*EPAS1*	Endothelial PAS domain protein 1	+	+	–	–	Angiogenesis, mitochondrion, response to hypoxia
*ERG*	ETS transcription factor-related gene	+	+	+	+	Cell differentiation
*FADD*	Fas associated via death domain	+	–	–	–	Apoptosis, pathways in cancer
*FGFR2*	Fibroblast growth factor receptor 2	+	+	–	+	Angiogenesis, Pathways in cancer
*FOXO1*	Forkhead box O1	+	+	–	–	Apoptotic process, response to hyperoxia
*GRB2*	Growth factor receptor bound protein 2	+	–	–	–	MAPK signaling pathway, insulin signaling pathway, pathways in cancer
*HK2*	Hexokinase 2	+	+	–	–	HIF-1 signaling pathway
*ICA*	Porcine inhibitor of carbonic anhydrase	+	+	+	–	HIF-1 signaling pathway
*IGF1R*	Insulin-like growth factor 1 receptor	+	+	–	–	Apoptosis process, HIF-1 signaling pathway
*IL6R*	Interleukin 6 receptor	+	–	–	–	HIF-1 signaling pathway, PI3K-Akt signaling pathway
*KIT*	KIT proto-oncogene receptor tyrosine kinase	+	+	–	–	Inflammatory response, ATP binding, pathways in cancer
*MAP2K1*	Mitogen-activated protein kinase 1	+	–	+	–	HIF-1 signaling pathway, VEGF signaling pathway
*SMAD2*	SMAD family member 2	+	–	–	–	TGF-beta signaling pathway
*STAT5B*	Signal transducer and activator of transcription 5B	+	–	–	–	Apoptosis process, erythrocyte differentiation, pathways in cancer

Note: “+” represents differentially methylated genes in some comparisons. “-” represents no differentially methylated genes. TH, Tibetan highland pig; YH, Yorkshire highland pig; TL, Tibetan lowland pig; YL, Yorkshire lowland pig

## Discussion

DNA methylation, an epigenetic modification, is an essential regulator of gene expression. Patterns of DNA methylation are determined in early development and are heritable [35, 36]. Although DNA methylation is stably maintained during cell division, it can be dynamically changed in response to the environment [37]. A complete characterization of the methylome and its dynamic changes may serve for an accurate disease prognosis [38]. In the present study, we found that there were differences in DNA methylation between Tibetan and Yorkshire pigs as well as between highland and lowland pigs within the same breed. The patterns of methylation in highland Tibetan pigs may represent regulatory mechanisms for adaptation to high-altitude hypoxia.

Tibetan pigs, which have a long history of living at high altitude and have experienced strong selection, have a well-developed heart, and are well adapted to low oxygen conditions [22]. Immigrant mammals that migrate to high altitudes are exposed to chronic hypoxia, which would result in cardiac hypertrophy and eventual heart failure [39, 40]. We believe that the 384 DMGs identified by comparing TH vs. YH, were of the most interest as potential candidate genes for high-altitude adaptation in Tibetan pigs. Of these 384 DMR, 183 were also identified by comparing TL vs. YL and thus represented stable differences in methylation between the two breeds. However, the 80 DMGs identified by comparing TH vs. TL, could be hypoxia-responsive genes in Tibetan pigs, while the 40 DMGs identified by comparing YH vs. YL could be hypoxia-responsive genes in Yorkshire pigs.

Adaptation to hypoxia is a complex process that involves multiple genes and various pathways [28]. Previous research has demonstrated that intermittent hypoxia may initiate epigenetic changes leading to long-lasting increases in oxidative stress and eventually to manifestations of cardiovascular disease in adult rats, while excessive hypoxic exposure in adult rats may induce the early onset of autonomic dysfunction, caused by DNA hypermethylation [41]. Additionally, DNA methylation has been shown to be involved in hypoxic adaptation in Tibetan chicken embryos [42].

HIF-1, which is a pivotal transcription factor, has numerous target genes that regulate the processes of cell proliferation, angiogenesis, glucose metabolism, and apoptosis in response to hypoxia [43–45]. In this study, eight DMGs, including *IGF1R* and *AKT3*, were enriched in the HIF-1 signaling pathway. The *IGF1R* gene is responsible for cell proliferation and survival and is expressed in many types of cancer cells [46]; enhanced activation of *IGF1R* is also implicated in the resistance to chemotherapy in a hypoxic microenvironment [47–49]. Previous studies have shown that *AKT3* plays an important role in cell proliferation, migration, and survival [50–53] and have revealed that it is also involved in the regulation of estrogen receptor-binding fragment-associated antigen 9 (*EBAG9*) and vascular endothelial growth factor (VEGF) secretion in ovarian cancer cells [54]. The *AKT3* expression could prevent the impaired angiogenesis in endothelial progenitor cells [55], which suggested that *AKT3* played a functional role in promoting angiogenesis and vascular permeability by regulating *VEGF* [56]. Therefore, the high levels of expression and low levels of methylation of *IGF1R* and *AKT3* in the Tibetan pig may trigger changes in their expression, potentially leading to adaptation to hypoxic environments.

Some other noteworthy DMGs, including *EPAS1, FGFR2, FOXO1,* and *SMAD2*, were found in this study. A genome-wide scanning revealed that *EPAS1* and the other genes had strong selection signals in Tibetans [57–62]. Depletion of *FGFR2* in cancer cells attenuated the hypoxia-mediated cell invasion [63], while forkhead box O1 (*FOXO1*) expression increased in smooth muscle cells and cardiomyocytes under hypoxia [64, 65].

## Conclusions

Our findings provide a comprehensive, detailed picture of DNA methylation patterns and distribution under hypoxic adaptation. The MeDIP-seq data identified, with sufficient depth and high resolution, genome- wide spanned DMRs, suggesting that this technique represents an effective approach for DNA methylome analysis. The genes modified by DNA methylation for adaptation to high-altitude in Tibetan pig may be involved in the HIF-1, insulin, and VEGF signaling pathways. We identified 19 DMGs that are potentially related to hypoxic adaptation in Tibetan pig. The present study provides new insights into hypoxic adaptation and its relationship to DNA methylation.

## Additional files

Additional file 1: Primers for bisulfite sequencing PCR. (XLSX 11 kb)

Additional file 2: Primers for qPCR. (XLSX 11 kb)

Additional file 3: Summary of sequencing reads obtained by MeDIP-seq. (XLSX 11 kb)

Additional file 4: **Figure S1.** Distribution of peaks varied with peak length in the four groups of pigs. **Figure S2.** Distribution of peaks in different gene elements in the four comparsion groups. The X-axis indicates different gene elements, and the Y-axis indicates the number of peaks. **Figure S3.** Distribution of DMRs in different gene elements in the four groups of pigs. **Figure S4.** Distribution of DMRs in CpG island, open sea, shelf, and shore regions. **Figure S5.** Venn diagram of differentially methylated genes (DMGs) among the four comparison groups. TH, Tibetan highland pig; TL, Tibetan lowland pig; YH, Yorkshire highland pig; YL, Yorkshire lowland pig. **Figure S6.** Validation of differentially methylated or differentially expressed genes by bisulfite sequencing and qPCR, respectively. (a–e) Methylation of CpG dinucleotide in *BCKDHB, EPHX2, GOT2, RXRG*, and *UBD*. (f) qPCR results for the five DMGs. (PDF 1380 kb)

Additional file 5: DMRs and DMGs in the four groups of pigs. (XLSX 3191 kb)

Additional file 6: qPCR and MeDIP-seq results. (XLSX 9 kb)

Additional file 7 GO functional analysis of DMGs in the four groups of pigs. (XLSX 24 kb)

Additional file 8 KEGG pathway analysis of DMGs in the four groups of pigs. (XLSX 13 kb)

## Abbreviations

AKT: Protein kinase B
AKT3: AKT serine/threonine kinase 3
AMPK: AMP-activated protein kinase
ANGPT2: Angiopoietin 2
BCKDHB: Branched-chain keto acid dehydrogenase E1 subunit beta
BSP: Bisulfite sequencing polymerase chain reaction
CAT: Catalase
CD38: Cluster of differentiation 38
CDS: Coding sequence
CGI: CpG island
DMG: Differentially methylated gene
DMR: Differentially methylated region
EBAG9: Estrogen receptor-binding fragment-associated antigen 9
EIF4E2: Eukaryotic translation initiation factor 4E family member 2
EPAS1: Endothelial PAS domain protein 1
EPHX2: Epoxide hydrolase 2
ERG: ETS transcription factor-related gene
FADD: Fas associated via death domain
FGFR2: Fibroblast growth factor receptor 2
FOXO1: Forkhead box O1
GO: Gene Ontology
GOT2: Glutamic-oxaloacetic transaminase 2
GRB2: Growth factor receptor bound protein 2
HIF-1: Hypoxia-inducible factor 1
HK2: Hexokinase 2
HPRT: Hypoxanthine phosphoribosyltransferase
ICA: Inhibitor of carbonic anhydrase
IGF1R: Insulin-like growth factor 1 receptor
IL6R: Interleukin-6 receptor
KEGG: Kyoto Encyclopedia of Genes and Genomes
KIT: KIT proto-oncogene receptor tyrosine kinase
MAP2K1: Mitogen-activated protein kinase 1
MeDIP-seq: Methylated DNA immunoprecipitation sequencing
NFAT: Nuclear factor of activated T cells
PI3K: Phosphoinositide 3-kinase
PPAR: Peroxisome proliferator-activated receptor
qPCR: Real-time fluorescence quantitative polymerase chain reaction
RXRG: Retinoid X receptor gamma
SMAD2: SMAD family member 2
STAT5B: Signal transducer and activator of transcription 5B
TH: Tibetan highland pig
TL: Tibetan lowland pig
TTR: Transcriptional termination region
UBD: Ubiquitin D
UTR: Untranslated region
VEGF: Vascular endothelial growth factor
YH: Yorkshire highland pig
YL: Yorkshire lowland pig
