# Supplementary material for: Genome-wide DNA methylation profiles in Tibetan and Yorkshire pigs under high-altitude hypoxia
Source: J Anim Sci Biotechnol. 2019 Feb 5;10:25. doi: 10.1186/s40104-019-0316-y (PMC6397503; doi:10.1186/s40104-019-0316-y)
Supplement: Supplementary file 4 — Figure S1. Distribution of peaks varied with peak length in the four groups of pigs. Figure S2. Distribution of peaks in different gene elements in the four comparsion groups. The X-axis indicates different gene elements, and the Y-axis indicates the number of peaks. Figure S3. Distribution of DMRs in different gene elements in the four groups of pigs. Figure S4. Distribution of DMRs in CpG island, open sea, shelf, and shore regions. Figure S5. Venn diagram of differentially methylated genes (DMGs) among the four comparison groups. TH, Tibetan highland pig; TL, Tibetan lowland pig; YH, Yorkshire highland pig; YL, Yorkshire lowland pig. Figure S6. Validation of differentially methylated or differentially expressed genes by bisulfite sequencing and qPCR, respectively. (a–e) Methylation of CpG dinucleotide in BCKDHB, EPHX2, GOT2, RXRG, and UBD. (f) qPCR results for the five DMGs. (PDF 1380 kb) [file 40104_2019_316_MOESM4_ESM.pdf]

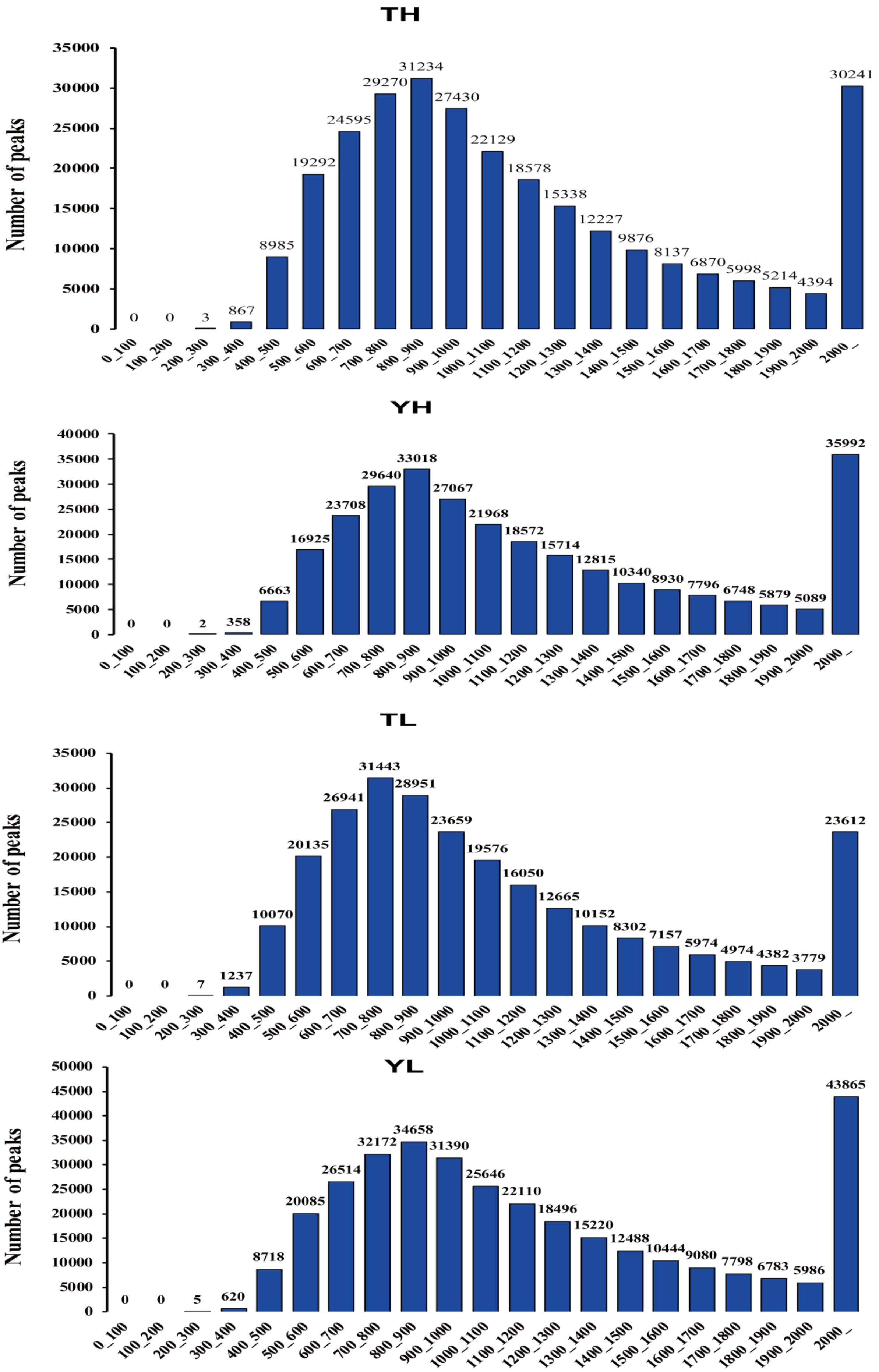

Figure S1 Distribution of peaks varies with peak length in four groups. The X-axis indicates the range of peak length, the Y-axis indicates the number of peaks.

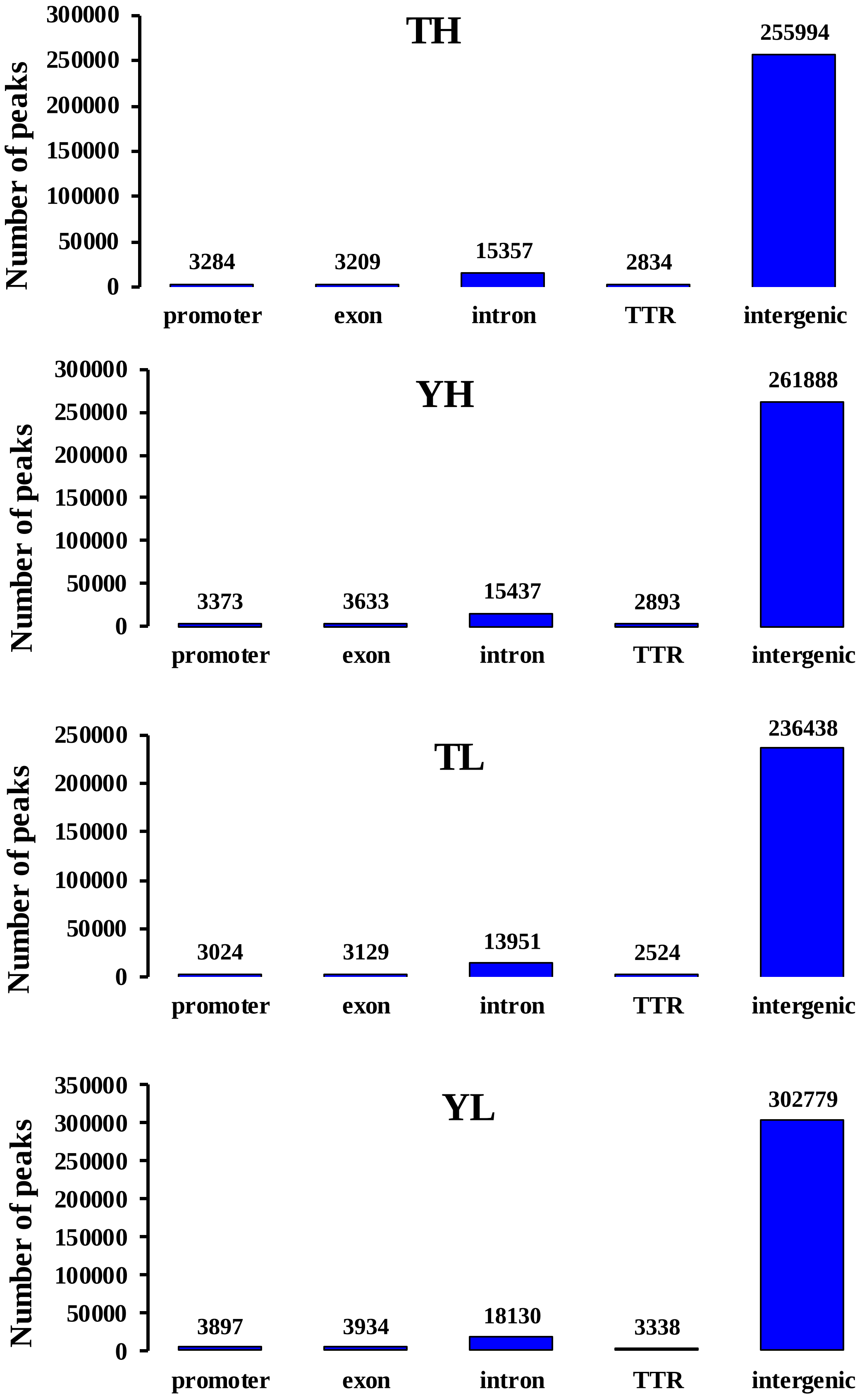

Figure S2 Distribution of peaks in different gene elements in the four comparsion groups. The X-axis indicates different gene elements, and the Y-axis indicates the number of peaks.

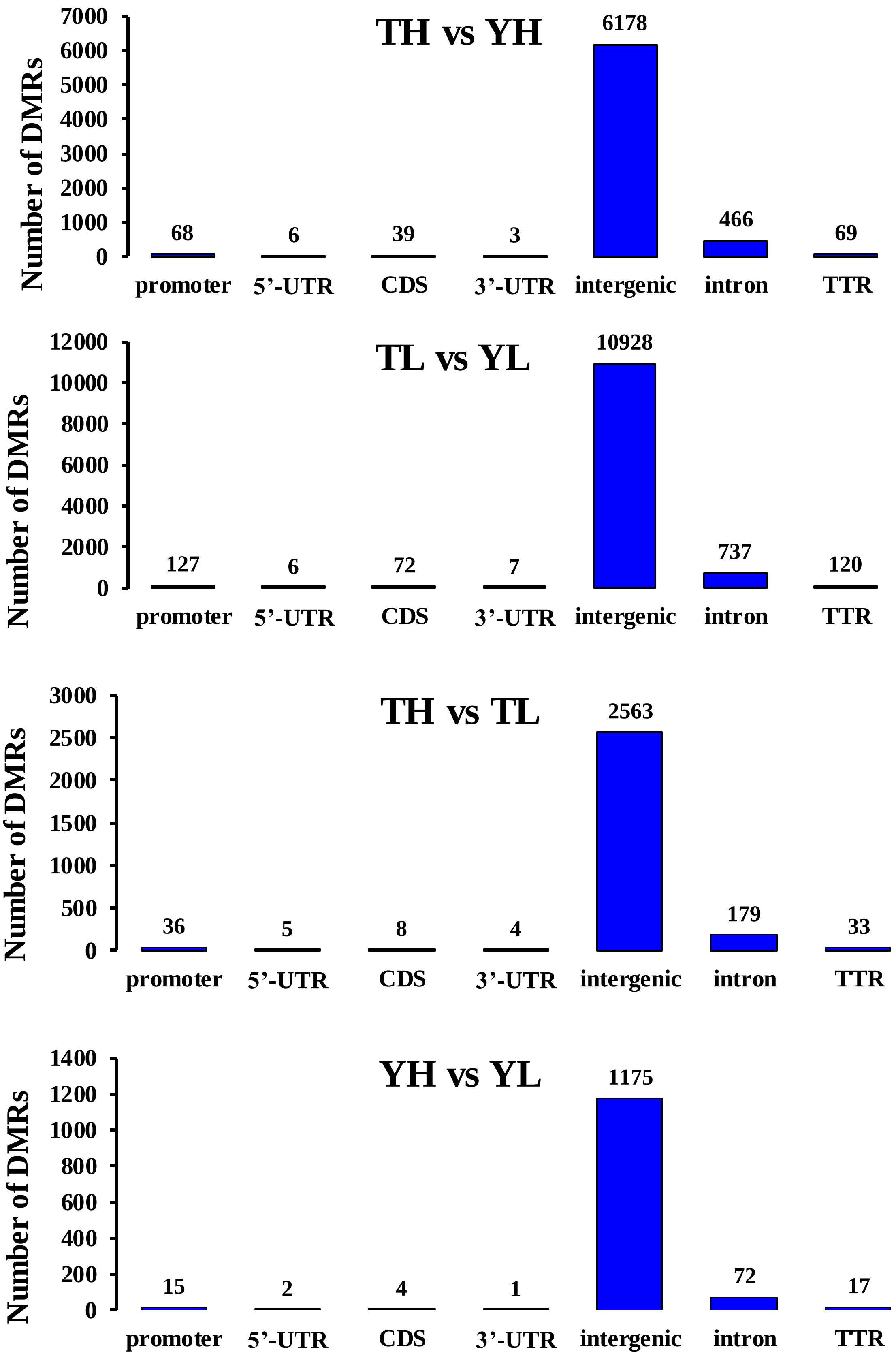

Figure S3 Distribution of DMRs in different gene elements in the four comparsion groups. The X-axis indicates different gene elements, and the Y-axis indicates the number of DMRs.

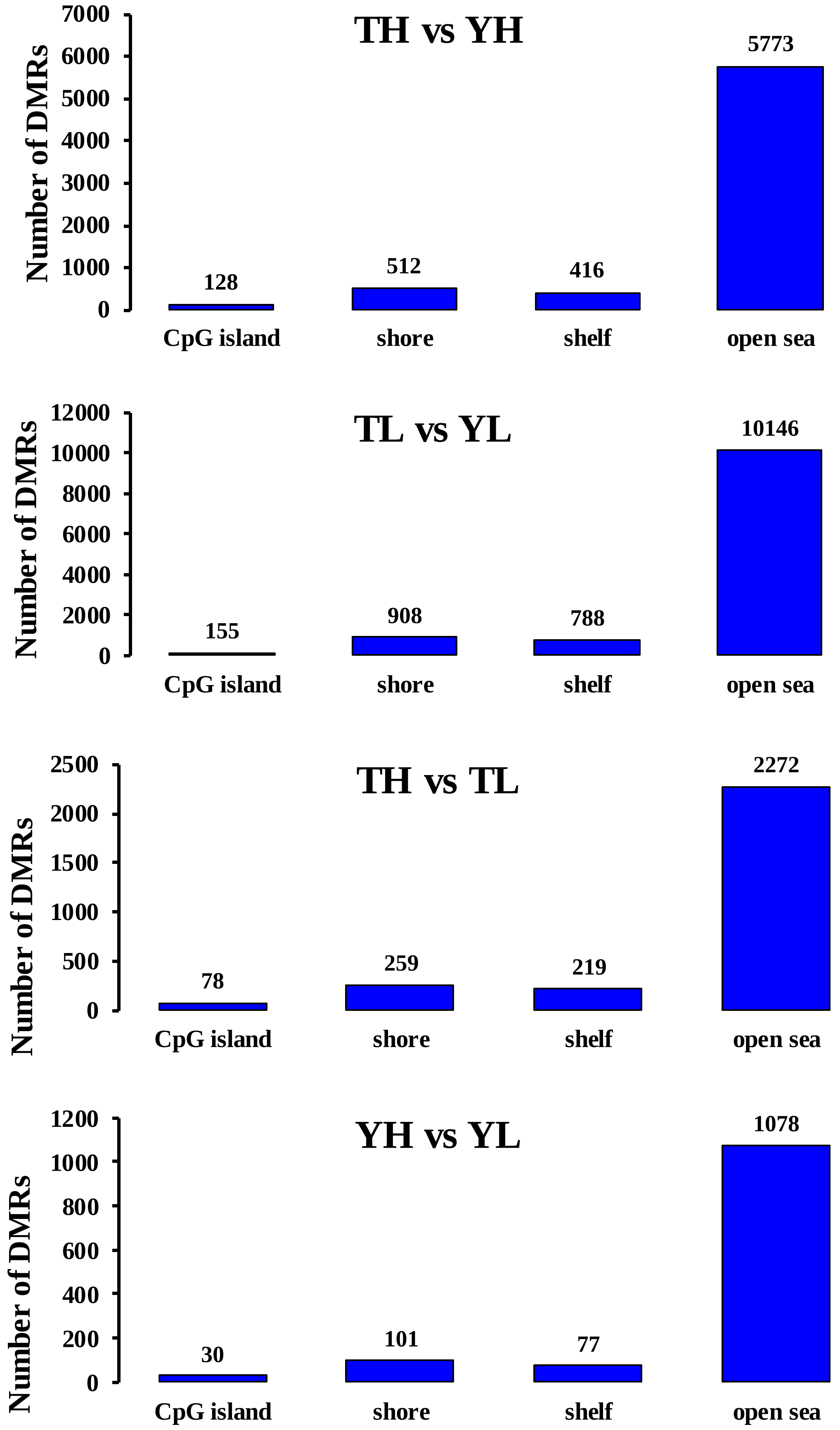

Figure S4 Distribution of DMRs in CpGIsland,open sea,shelf and shore. The X-axis indicates different gene elements, and the Y-axis indicates the number of DMRs.

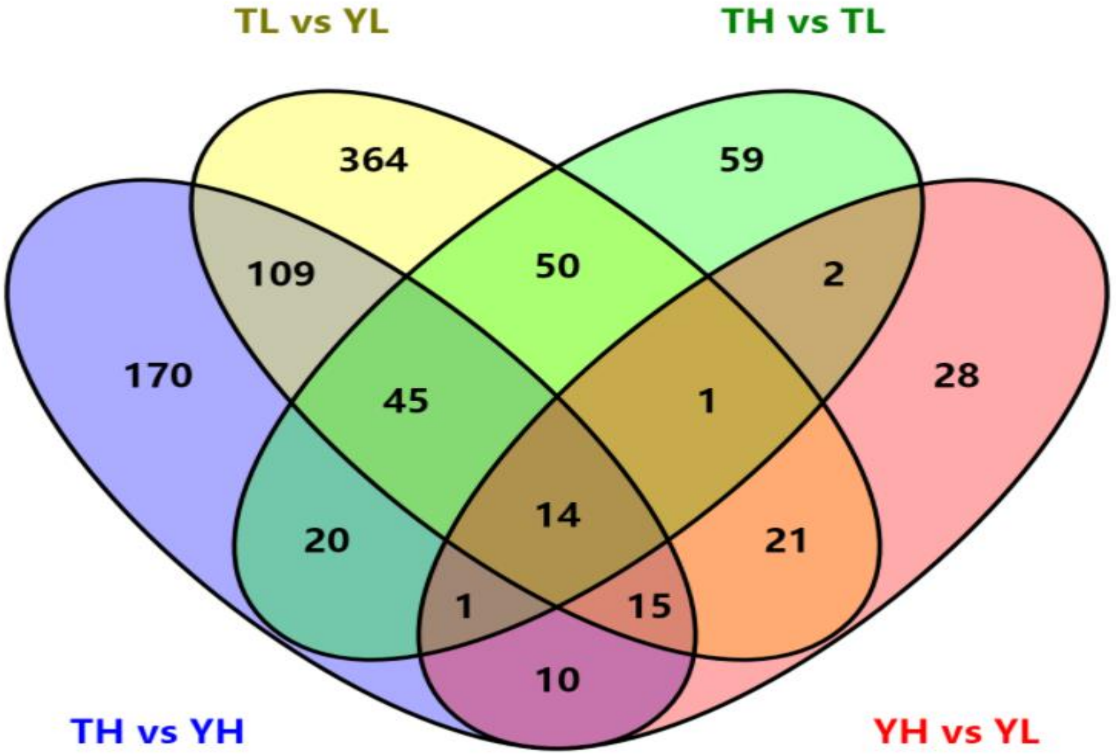

Figure S5 Venn diagram of differentially methylated genes (DMGs) among the four comparison groups. TH, Tibetan highland pig; TL, Tibetan lowland pig; YH, Yorkshire highland pig; YL, Yorkshire lowland pig.

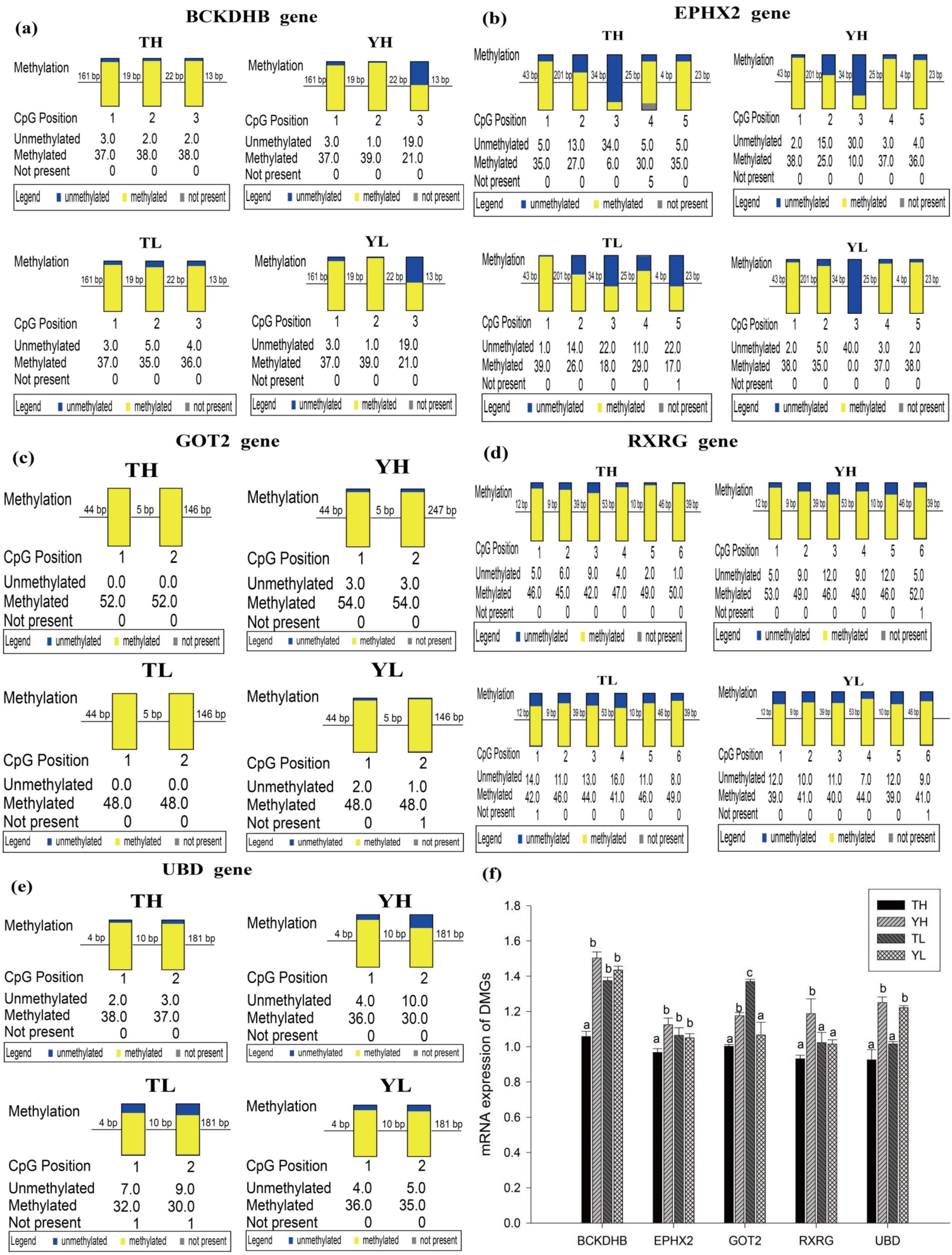

Figure S6 Validation of differentially methylated or differentially expressed genes

by bisulphite sequencing and qPCR, respectively. (a–e) Methylation of CpG dinucleotide in BCKDHB, EPHX2, GOT2, RXRG and UBD, respectively. The different columns represent different CpG sites, bp numbers in the middle represent the distance between two adjacent CpG sites, the yellow column represents the methylation site, the blue represents the unmethylation site, and the gray represents the deletion site. The number of clones in each state is indicated below each column. **(f)** qPCR results of five DMGs. Upper letters (a, b and c) on bars denote significantly different expression levels in the same gene, the same letter indicates no significant difference ($P > 0.05$), different letters indicate significant differences ($P < 0.05$).
